# Bioimaging of Lysosomes with a BODIPY pH-Dependent Fluorescent Probe

**DOI:** 10.3390/molecules27228065

**Published:** 2022-11-20

**Authors:** Raquel C. R. Gonçalves, Efres Belmonte-Reche, João Pina, Milene Costa da Silva, Sónia C. S. Pinto, Juan Gallo, Susana P. G. Costa, M. Manuela M. Raposo

**Affiliations:** 1 Centre of Chemistry, University of Minho, Campus de Gualtar, 4710-057 Braga, Portugal; 2 Advanced (Magnetic) Theranostic Nanostructures Lab, International Iberian Nanotechnology Laboratory, Av. Mestre José Veiga s/n, 4715-330 Braga, Portugal; 3 Coimbra Chemistry Centre-Institute of Molecular Sciences, Department of Chemistry, University of Coimbra, 3004-535 Coimbra, Portugal

**Keywords:** BODIPY, synthesis, bioimaging, fluorescent probe, lysosomes, pH

## Abstract

Fluorescence-based probes represent a powerful tool for noninvasive imaging of living systems in real time and with a high temporal and spatial resolution. Amongst several known fluorophores, 3-difluoroborodipyrromethene (BODIPY) derivatives have become a cornerstone for innovative fluorescent labelling applications, mainly due to their advantageous features including their facile synthesis, structural versatility and exceptional photophysical properties. In this context, we report a BODIPY-based fluorescent probe for imaging of lysosomes in living cells. The BODIPY derivative displayed a remarkable fluorescence enhancement at low pH values with a pK_a_* of 3.1. In vitro studies by confocal microscopy in HeLa cells demonstrated that the compound was able to permeate cell membrane and selectively label lysosome whilst remaining innocuous to the cell culture at the maximum concentration tested. Herein, the BODIPY derivative holds the promise of investigating lysosomal dynamics and function in living cells through fluorescence imaging.

## 1. Introduction

During the past decades there has been significant interest in the design and synthesis of organic chromophores with strong absorption and fluorescence to meet the demand in bioimaging techniques for biological and medicinal applications. As a matter of fact, optical imaging, which includes fluorescence-based imaging, can provide a highly sensitive and selective detection of the signal emitted from fluorophores at different wavelengths, allowing the simultaneous imaging of multiple fluorescent probes [1,2,3].

Hence, fluorescent probes have enabled the characterization of dynamic cell mechanisms as well as the monitorization of physiological environment variations that may be causes or symptoms of several health disorders. These include, for example, intracellular pH variation and intracellular heavy metal ion accumulation [4,5,6,7].

Amongst the different types of organic fluorescent probes, 3-difluoroborodipyrromethene (BODIPY) derivatives are demonstrating a growing success in the bioimaging field due to their appropriate photophysical properties and great photostability in biological conditions. This enables an efficient fluorescence signal detection during imaging acquisition. Besides, their facile synthesis and functionalization allows the modulation of their absorption/emission profiles and the introduction of recognition sites/moieties to specifically target biological relevant species such as biomolecules, inorganic bioanalytes and cellular organelles [8,9,10,11,12]. In this context, several strategies to design specific targeting-probes have been proposed [13,14,15], including for the lysosome, which is a vesicular acidic organelle involved in processes related to digestion of biomolecules (e.g., proteins, lipids, carbohydrates) and defense against pathogenic viruses and bacteria [16,17]. Both these processes are mediated by hydrolytic enzymes, whose function is most efficient in low pH environments [18,19]. Additionally, the difference of the lysosome’s pH with regard to the cellular cytoplasm’s (pH 4.5–6.0 vs. pH ~7.2, respectively) is further exacerbated in cancer cells, where lysosomal pH can become more acidic (pH 3.8–4.7) because of the higher expression of specific subunits that promote the activity of V-ATPase proton pump [20,21]. Having this in mind, pH-conditioned fluorescence has been a major consideration for the development of probes designed to investigate the lysosomal morphology and functions [22,23].

Among others, benzimidazole-based molecules have been reported as fluorescence probes for pH sensing. Due to its inherent features, the benzimidazole moiety has often been incorporated in fluorophore cores to grant the final adduct the pH-dependent fluorescence emission derived from its amino protonation and deprotonation state [24,25].

Herein, the present work reports the synthesis of a *meso* anthracene-BODIPY derivative functionalized with a benzimidazole moiety at position 2 of the BODIPY core. The study of the pH-dependent absorption and emission features of the BODIPY derivative evidenced a remarkable fluorescence enhancement under acidic conditions. Furthermore, in vitro studies showed that the BODIPY derivative is innocuous to the cells, yet its fluorescence is triggered in the acidic lysosomal environment within cells, thus demonstrating its potential as fluorescent probe for live-cell imaging of lysosomes.

## 2. Results and Discussion

### 2.1. Synthesis and Characterization of BODIPY Derivative ***3***

**Scheme 1:**
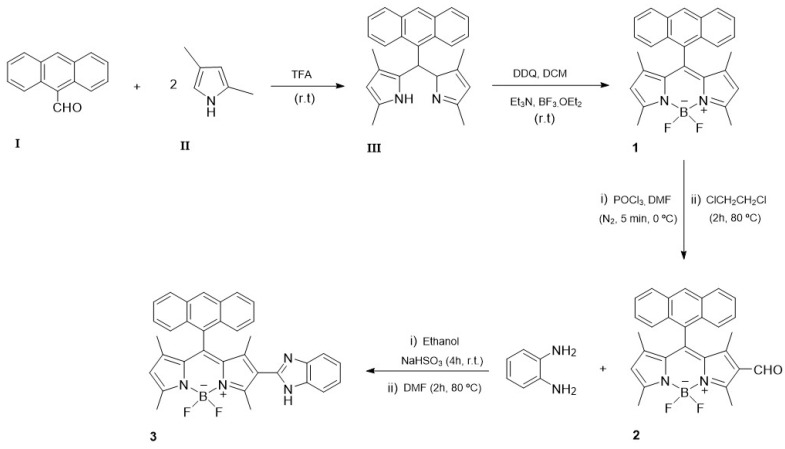
Synthetic route for BODIPY derivative **3** [26,27].

The synthesis of BODIPY derivative **3**, as well as the corresponding precursors **1**–**2** have recently been reported by our group [26,27] and by others [28,29]. The synthetic route for BODIPY derivative **3**, performed by our group, is represented in Scheme 1.

**Figure 1:**
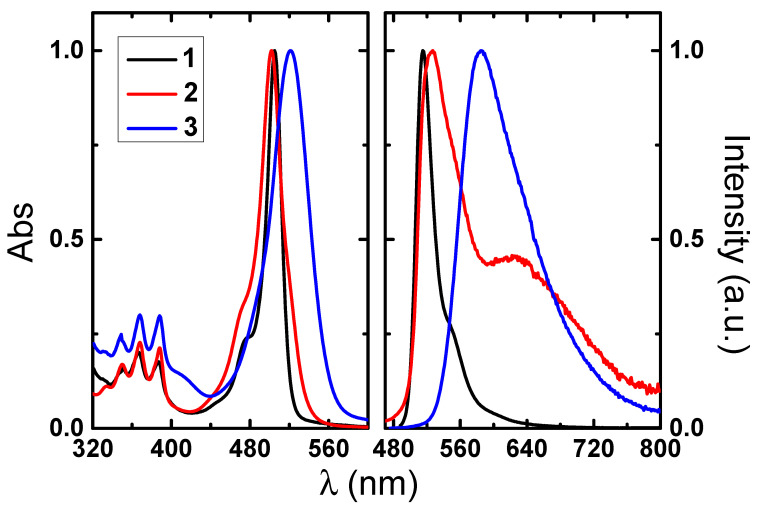
Absorption and fluorescence emission spectra for BODIPYs **1**–**3** in tetrahydrofuran solution at 293 K.

The symmetrical *meso*-substituted anthracene-BODIPY **1** was synthesized by Lindsey’s method [30,31,32,33]. The introduction of an aldehyde group at position 2 of the BODIPY core was achieved via Vilsmeier–Haack formylation to obtain the formyl-BODIPY precursor **2**. Finally, BODIPY derivative **3** functionalized with a benzimidazole group was synthesized through the condensation between the formyl group and *o*-phenylenediamine to yield the benzimidazole moiety. Characterization of the BODIPY precursors **1**–**2** and BODIPY **3** was performed through NMR and high-resolution mass spectrometry and the data were in accordance with those previously published. Concerning the photophysical properties, the absorption spectra of the investigated BODIPYs in THF solution, present the characteristic spectroscopic features of the anthracene chromophore at lower wavelengths (in the 320–400 nm range) and the BODIPY strong absorption band with maximum at ∼502–520 nm (see Figure 1 and Figure S1) [12,31]. The fluorescence emission spectra of BODIPY **1**–**3** in THF solution collected with excitation at 360 nm (where the anthracene chromophore mainly absorbs) and at the BODIPY absorption band, always displays the characteristic BODIPY emission band with maximum at ~515–585 nm (Figure 1), thus showing that efficient energy transfer occurs between the anthracene and the BODIPY moieties. The good agreement between the BODIPY **1** absorption spectra and the fluorescence excitation spectra collected at 555 nm, which reproduces the spectroscopic features of the chromophoric units, offers further support of the occurrence of energy transfer, see Figure S1.

The emission spectra of the formyl-BODIPY **2** in THF presents an additional broad and structureless red-shifted emission band when compared to the characteristic BODIPY emission spectra (see Figure 1), promoted by the presence of the electron-withdrawing formyl group and attributed to intramolecular charge transfer (ICT). The electron-withdrawing behavior of the formyl group in BODIPY **2** in THF solution is supported by the observed decrease in the fluorescence quantum yield going from BODIPY **1** to **2** (ϕ_F_ = 0.43 vs. 0.02, respectively, see Table 1).

Moreover, the ICT character in the excited-state of both BODIPY **1** and **2** was enhanced with the more polar solvent acetonitrile, as shown: (i) by the appearance of a CT emission band at lower energies for BODIPY **1** and the bathochromic shift of BODIPY **2** CT band (see Figure S2); and (ii) by the decrease in the fluorescence quantum yields values going from THF to acetonitrile (ϕ_F_ = 0.43 vs. 0.03 for BODIPY **1** and ϕ_F_ = 0.02 vs. 0.01 for BODIPY **2**, see Table 1).

When comparing the spectroscopic data of BODIPY **3** with its precursors **1**–**2**, a red-shifted absorption and emission maximum was observed, along with a larger Stokes-shift (Δ_SS_ = 2100 cm^−1^ vs. 385 cm^−1^ and 835 cm^−1^, respectively) (Table 1). The high Stokes-shift value, together with the positive solvatochromic effect and the decrease in the ϕ_F_ value going from THF to acetonitrile (ϕ_F_ = 0.35 vs. 0.10, respectively, Table 1 and Figure S2) supports the occurrence of charge transfer in BODIPY **3**; thus, in agreement with what was previously reported for benzimidazole-BODIPY derivatives, which showed that photoinduced electron transfer occurs from the benzimidazole moiety to the BODIPY chromophore [31,34]. The strong fluorescence emission (ϕ_F_ = 0.35) combined with the large Stokes-shift are desirable features for biological imaging applications of BODIPY **3**. Indeed, a large Stokes-shift avoids spectral overlap between absorption and emission, allowing detection of a stronger fluorescence signal while reducing interference.

**Figure 2:**
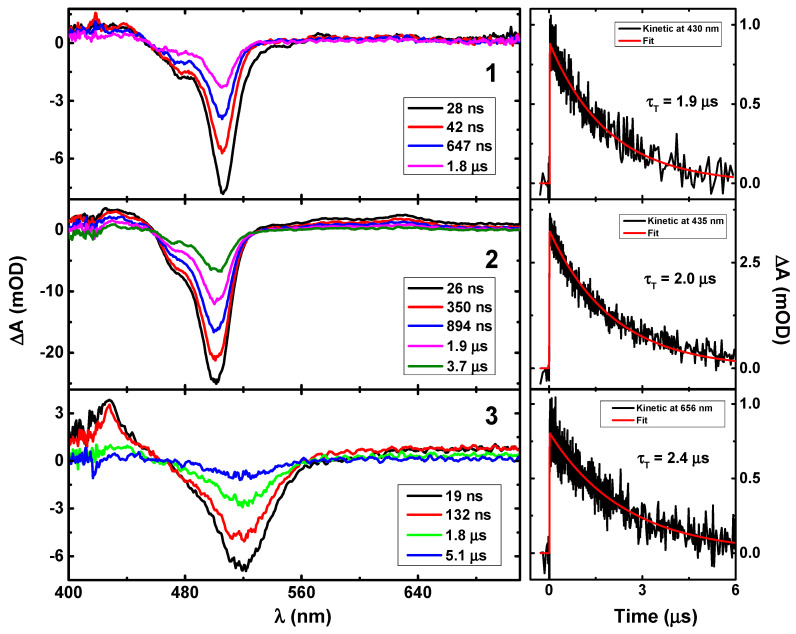
Room temperature time-resolved triplet-singlet difference absorption spectra together with the characteristic kinetic traces and triplet lifetimes for BODIPY **1**–**3** in deaerated tetrahydrofuran solutions collected with excitation at 370 nm.

The triplet-triplet transient absorption spectra of deaerated solutions of the BODIPY derivatives were collected by nanosecond-millisecond pump-probe transient absorption spectroscopy. Similar transient triplet-triplet absorption spectra and triplet lifetimes (τ_T_ ~ 2 μs) were found for the investigated BODIPY derivatives in THF solution (Figure 2). The transient absorption spectra of BODIPY **1**–**3** present a broad positive triplet-triplet absorption band in the 400–460 nm and 540–700 nm ranges, and negative strongly overlapped bands resulting from bleaching of the ground state absorption. To estimate the intersystem crossing quantum yields (triplet state formation quantum yield, ϕ_T_) we measured the singlet oxygen sensitization quantum yields (ϕ_Δ_) for BODIPY **1**–**3**, since ϕ_Δ_ is proportional to the triplet state formation quantum yield (assuming efficient ^1^O_2_ sensitization, S_Δ_ *=* ϕ_Δ_/ϕ_T_ ≈ 1) [35]. BODIPY **3** presents the lowest singlet oxygen sensitization quantum yield value (ϕ_Δ_ = 0.02 vs. 0.29 and 0.36 for **1** and **2**, respectively, Table 1), which could anticipate a low phototoxicity in cells.

From the quantum yields in Table 1 (ϕ_Δ_/ϕ_T_ ≈ 1) together with the internal conversion quantum yield (ϕ_IC_ = 1 − ϕ_F_ − ϕ_T_) we can conclude that: (i) for BODIPY **1** the radiative deactivation is the main excited-state decay channel (ii) while for BODIPY **2** and **3** the radiationless processes (ϕ_T_ + ϕ_IC_) prevail in the excited state decay (with ϕ_IC_ ≈ 0.61).

### 2.2. pH-Dependent Absorption and Fluorescence Spectra

**Figure 3:**
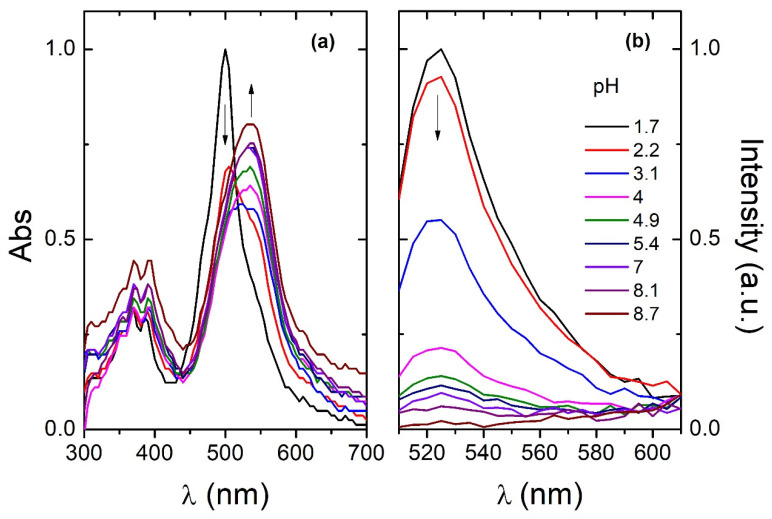
(**a**) Absorption and (**b**) fluorescence spectral changes of BODIPY **3** under different pH conditions (pH = 1.7–8.7).

**Figure 4:**
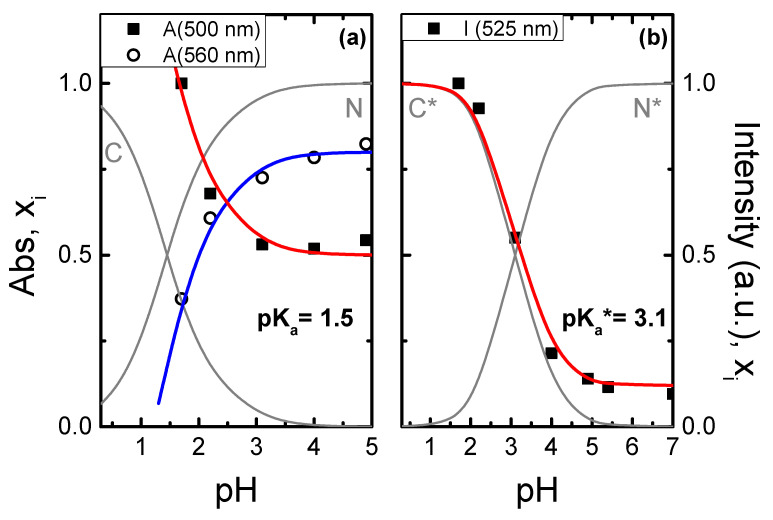
(**a**) Absorption and (**b**) steady-state fluorescence emission titration curves for BODIPY **3** together with the best fits to the data. The absorption and emission intensities were normalized to be superposed to the molar fraction distribution of species, x_i_, the ground- and excited-state protonated (cationic, C) and neutral (N) species.

The pH dependency of the absorption and fluorescence properties of BODIPY **3** were investigated whilst considering the protonation properties ascribed to the benzimidazole moiety at position 2 of the BODIPY core [24]. Figure 3a shows the BODIPY’s absorption spectra in aqueous solutions at different pH values, from 1.7 to 8.7. As it is appreciable in the spectra, the absorption peak centered at 500 nm decreased as the pH values increased. However, simultaneous to this decrease, a new band with a maximum at 540 nm increased with pH. This significant red-shift in the maximum absorption band can be attributed to the deprotonation of the benzimidazole NH. In agreement with previous reports [34], the absorption spectra of BODIPY **3** in the investigated pH range, presents the acid-base equilibria involving the protonated (cationic) and neutral species with maxima at 500 nm and 540 nm, respectively. From the spectrophotometric titration, a ground-state dissociation constant (pKa) value of 1.5 was obtained (Figure 4a), which was lower than those previously reported for benzimidazole derivatives (pKa values of ~5) [34,36].

In the fluorescence spectra, an intense emission band at 525 nm was obtained at pH 1.7, yet at higher pH values, a significant fluorescence quenching effect was observed as shown in Figure 3b. From the fluorescence emission titration (Figure 4b), the excited state dissociation constant for the equilibrium between the protonated and neutral species was found to be pKa* = 3.1, thus demonstrating a remarkable fluorescence emission response of BODIPY derivative **3** to acidic pH levels. Altogether, the results indicate the derivative’s potential application as a fluorescent acidic probe.

### 2.3. Cell Viability

**Figure 5:**
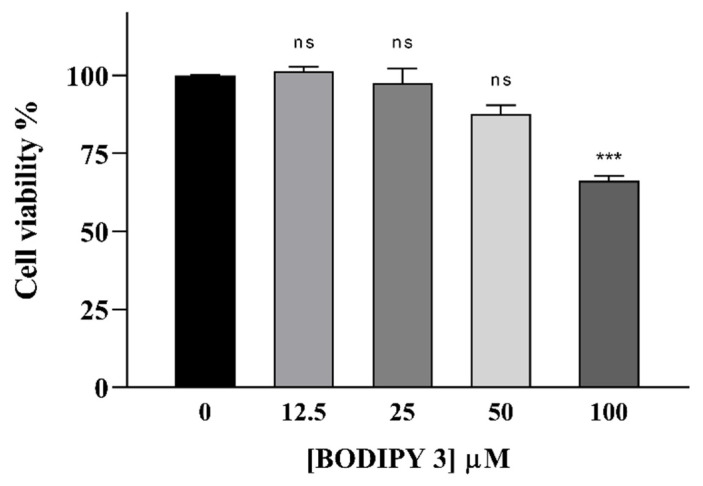
Viability assay in HeLa cells. Cells were treated with different concentrations of BODIPY **3** for 24 h. The cell viability was measured using resazurin assay and data are presented as mean ± SEM from triplicate samples of two independent experiments. One-way ANOVA followed by Dunnett’s multiple comparisons test was used to determine the level of significance based on *p* > 0.05 (ns) and *p* = 0.0008 (***) compared to the control group.

Due to the higher fluorescence intensity of BODIPY **3** at lower pH values, its bioimaging application as a fluorescent probe of acidic organelles, namely, lysosomes, was explored. Prior to cell imaging, BODIPY **3** cytotoxicity was evaluated in HeLa cells using the reference resazurin assay. As shown in Figure 5, cell viability after treatment with compound **3** remained greater than 87% with concentrations of 50 µM after 24 h. When incubated with 100 µM, the viability of cells did not reach the compound’s IC_50_ value (viability of 66%), indicating the compound’s innocuousness even at this large concentration. Still, a concentration of 20 µM of BODIPY was used to ensure biocompatibility and negligible cytotoxic effect on HeLa cells whilst performing live-imaging experiments.

### 2.4. Live-Imaging

#### Lysosome Colocalization Assay

**Figure 6:**
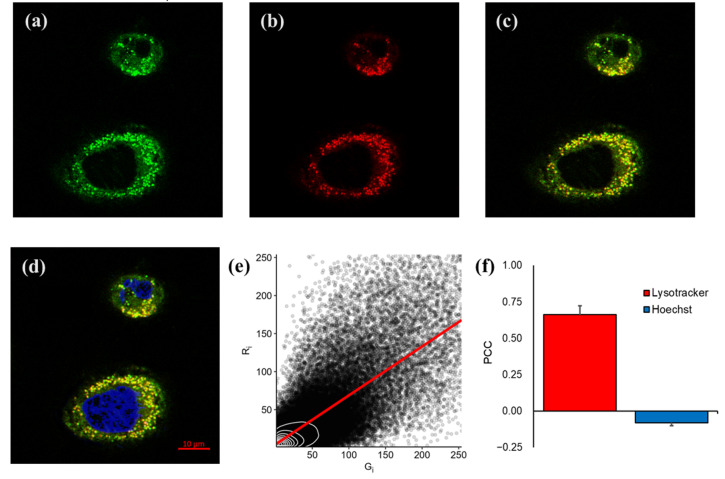
(**a**) Confocal fluorescence images of HeLa cells treated with BODIPY **3** and (**b**) Lysotracker Deep Red. (**c**) Merge of BODIPY fluorescence signal (green channel, λ_ex_ 488 nm) and Lysotracker fluorescence signal (red channel, λ_ex_ 633 nm). (**d**) Merge of Hoechst 33342 fluorescence signal (blue channel, λ_ex_ 405 nm), BODIPY **3** fluorescence signal (green channel) and Lysotracker fluorescence signal (red channel). (**e**) Scatterplot of the pixel intensity for red (R_i_) and green (G_i_) channels. (**f**) Pearson’s correlation coefficient (PCC) of BODIPY with Lysotracker and Hoechst 33342 dye.

To determine the internalization and localization of BODIPY **3** within HeLa cells, colocalization experiments by confocal microscopy were performed with a commercial lysosome fluorescent probe (LysoTracker Deep Red). In Figure 6a,b the fluorescence signal distribution of BODIPY **3** and Lysotracker for the green (λ_ex_ 488 nm) and red (λ_ex_ 633 nm) channels, respectively, are shown. Here, the BODIPY was able to rapidly cross the cell membrane and exhibited a bright and efficient intracellular fluorescence emission. As can be observed from the merged image of both channels (Figure 6c), the overlap between the green and red channel fluorescence results in a yellow signal that indicates BODIPY **3** and Lysotracker colocalization. In contrast, the merged image between the BODIPY **3** and the nucleus dye (Hoechst 33,342) demonstrated the absence of overlap (Figure 6d). Moreover, the scatterplot of the pixel intensity for red (R_i_) and green (G_i_) channels (Figure 6e) shows a clear positive linear correlation between the two variables. The Pearson’s correlation coefficient (PCC) was applied to measure the degree of colocalization, wherein a value of 1 indicates that the channel’s intensities are perfectly and linearly intertwined. The PCC value of BODIPY **3** with Lysotracker Deep Red was found to be 0.66 ± 0.06, which corroborates the correlation between BODIPY and Lysotracker. On the contrary, the PCC value calculated for BODIPY and Hoechst 33342 was near zero, reflecting uncorrelated signals between the two fluorescent dyes (Figure 6f).

These results suggest that BODIPY **3** is accumulating in lysosomes, where the pH environment is lower (∼pH 4.6) when compared to the cell cytoplasm (∼pH 7.4). Therefore, it is possible that BODIPY **3** molecules are diffusing through the cell membrane, to be captured later by these acidic organelles, and here, the benzimidazole moiety linked to the BODIPY core can protonate and activate the probe’s fluorescence emission, as expected from the previously described photophysical experiments.

Recently, Songrui Li et al. reported a fluorescent pH sensitive probe [37]. They used the probe to monitor intracellular pH variations in MCF-7 cells and determined that a significant decrease in emission at acidic pH occurred, contrary to what we found for the behavior of BODIPY **3**. Additionally, our compound exhibited a longer absorption wavelength that promotes a more efficient cell image acquisition by confocal microscopy. And also, the staining of lysosomes did not require cell fixation, which allows the visualization of biological dynamic processes in real-time. These features represent relevant advantages regarding BODIPY **3** application in imaging of living cells.

## 3. Materials and Methods

### 3.1. General

Materials and general instrumentation for the synthesis and characterization of the BODIPY derivatives **1**–**3** are described in Supporting Information.

The photophysical characterization of BODIPY derivative **3** and the corresponding precursors **1**–**2** was performed in tetrahydrofuran (THF) solutions. Absorption and fluorescence spectra were recorded on Shimadzu UV-2600i and Horiba-Jobin-Ivon Fluorolog 322 spectrometers, respectively. All the fluorescence emission spectra were corrected for the wavelength response of the system. Room temperature fluorescence quantum yields were obtained by the comparative method using rubrene in chloroform, ϕ_F_ = 0.54, as reference compound [38,39]. The experimental setup used to obtain the singlet-triplet difference spectra and triplet lifetimes consisted of a nanosecond-millisecond broadband (350–1600 nm) pump-probe Transient Absorption EOS-Fire spectrometer from Ultrafast Systems (Sarasota, FL, USA), pumped with an amplified femtosecond Spectra-Physics Solstice-100F laser (1 kHz repetition rate) coupled with a Spectra-Physics TOPAS Prime F optical parametric amplifier (195–22,000 nm). The transient absorption data were obtained with excitation 370 nm and probed in the 350–800 nm range. Low laser energy was used to avoid multiphoton and triplet-triplet annihilation effects. The solutions used to collect the transient singlet-triplet difference absorption spectra were bubbled with nitrogen for at least 20 min. Data analysis was performed using Surface Xplorer PRO program from Ultrafast Systems. In general, the obtained transient absorption signals were assigned to the triplet state of the BODIPY derivatives since first-order kinetics were found and strong quenching was observed in the presence of oxygen.

Room-temperature singlet oxygen NIR phosphorescence was detected with a Hamamatsu R5509-42 photomultiplier cooled to 193 K in a liquid nitrogen chamber (Products for Research model PC176TSCE-005) following (i) laser excitation at 355 nm of aerated THF solutions of the samples in an adapted Applied Photophysics flash kinetic spectrometer pumped with a Spectra-Physics Quantaray Nd:YAG laser, (ii) or using a Horiba-Jobin-Ivon Fluorolog 322 spectrometer (HJY322) [40]. In both cases, to avoid the overlap of the fluorescence emission second harmonic signal with the sensitized singlet oxygen phosphorescence emission band centered at 1276 nm, a Newport longpass dielectric filter with 1000 nm cut-on (reference 10LWF-1000-B) was used. The sample solutions were optically matched at the excitation wavelength (absorbances less than 0.3) with those of the reference compounds, 1H-phenalen-1-one, ϕ_Δ_ = 0.98 and tetraphenylporphyrin, ϕ_Δ_ = 0.60, in the polar solvent THF [41,42,43]. When using the Applied Photophysics setup the singlet oxygen sensitization quantum yield values (ϕ_Δ_) were determined by plotting the initial emission intensity of optically matched solutions as a function of the laser energy and comparing the slope with that obtained upon sensitization with the reference compound, see Equation (1):

(1) $$\phi_{\Delta }^{cp}=\frac{slope^{cp}}{slope^{ref}}\cdot \phi_{\Delta }^{ref}$$

where $\phi_{\Delta }^{ref}$ is the singlet oxygen formation quantum yield of the reference compound.

With the HJY322 spectrometer the ϕ_Δ_ values were obtained by collecting the sensitized phosphorescence emission spectra of singlet oxygen from optically matched solutions of the samples and that of the reference compound obtained in the same solvent and identical experimental conditions. The singlet oxygen formation quantum yield is then determined by comparing the integrated area under the emission spectra of the sample solutions,$\int I{\left(\lambda \right)}^{cp}d\lambda$, and that of the reference solution, $\int I{\left(\lambda \right)}^{ref}d\lambda$, and applying Equation (2),

(2) $$\phi_{\Delta }^{cp}=\frac{\int I{\left(\lambda \right)}^{cp}d\lambda }{\int I{\left(\lambda \right)}^{ref}d\lambda }\cdot \phi_{\Delta }^{ref}$$

### 3.2. Synthesis

The synthesis of BODIPY derivatives **1**–**3** have recently been reported by our group and by others [26,27,28,29]. Detailed information regarding the synthesis and complete characterization by our group can be found in the Supplementary Information.

### 3.3. pH-Dependent Absorption and Fluorescence Spectra

The evaluation of BODIPY derivative **3** as pH probe was performed in water at a pH range from 1.7 to 8.7. The solutions with different pH values were prepared in Milli-Q water by adding HCl (1 M) or NaOH (1 M) and the pH was determined with a pH meter. A stock solution of the BODIPY derivative was prepared in DMSO (10 mM) and then diluted in the aqueous pH solutions to a final concentration of 10 μM. The absorption and fluorescence intensities were monitored using a Biotek Synergy H1 plate reader (Agilent, Santa Clara, USA) (λ_exc_ = 480 nm). The ground-state (pK_a_) and excited-state dissociation constants (pK_a_*) were obtained by fitting the absorption and emission intensities (obtained from the characteristic bands of the species present in solution) together with their molar fraction distribution and the pK_a_ values. The molar fraction distributions were determined assuming the case of a monoprotic acid. In this case, following the deprotonation reaction below,

LH⇄L+H

the analytical concentration, *c_a_* can be given by,

(3) $$c_{a}=\left[LH\right]+\left[L\right]$$

Rearrangement of Equation (1) leads to:

(4) $$\frac{\left[LH\right]}{\left[LH\right]+\left[L\right]}+\frac{\left[L\right]}{\left[LH\right]+\left[L\right]}=x_{LH}+x_{L}$$

and considering that,

(5) $$k_{a}=\frac{\left[L\right]\left[H\right]}{\left[LH\right]}$$

the molar fraction distributions, x_i_, for the protonated (*LH*) and unprotonated (*L*) species, can be obtained from Equations (6) and (7):

(6) $$x_{LH}=\frac{\left[H\right]}{\left[H\right]+k_{a}}$$

(7) $$x_{L}=\frac{k_{a}}{\left[H\right]+k_{a}}$$

### 3.4. Cell Culture

Human cervical cancer (HeLa) cells were grown in Dulbecco’s modified Eagle’s medium (DMEM) (Gibco™, Thermo Fisher Scientific, Waltham, MA, USA) supplemented with 10% fetal bovine serum (FBS) and 1% of penicillin-streptomycin. Cells were maintained at 37 °C in a humidified incubator with a 5% CO_2_ atmosphere.

### 3.5. Cell Viability Assay

HeLa cell viability was determined by resazurin assay [44,45]. Furthermore, 10^4^ cells/well were seeded in 96-well plates and incubated overnight at 37 °C in a 5% CO_2_ atmosphere. Then, cells were incubated with BODIPY derivative **3** at concentrations ranging from 12.5 to 100 µM for 24 h. Afterwards, cells were treated with resazurin reagent (1:100) and incubated for 4 h in the dark. The plates were scanned in a Biotek Synergy H1 plate reader (λ_exc_ = 535 nm, λ_em_ = 595 nm). Cell viability was calculated considering the control (non-treated cells) as 100% viability. Data were represented as mean ± SEM from triplicate samples of two independent experiments. The statistical analysis was performed in GraphPad Prism 8.0.2 (GraphPad Software, San Diego, CA, USA) and data were analyzed using one-way ANOVA followed by Dunnett’s multiple comparisons test (ns: not significant (*p* > 0.05), *** *p* = 0.0008).

(8) $$\mathrm{Cell}\;\mathrm{viability}\;\%=\frac{\mathrm{Fluo}\;\left(\mathrm{treated}\;\mathrm{cells}\right)-\;\mathrm{Fluo}\;\left(\mathrm{blank}\right)\;}{\mathrm{Fluo}\;\left(\mathrm{control}\right)-\mathrm{Fluo}\;\left(\mathrm{blank}\right)}\times 100$$

### 3.6. Live-Imaging

#### 3.6.1. Lysosome Colocalization Assay

HeLa cells were seeded at a density of 2 × 10^4^ cells/well in growth medium in glass bottom dishes and incubated overnight at 37 °C in a 5% CO_2_ atmosphere. Then, cells were washed two times with PBS and treated with BODIPY derivative **3** at a concentration of 20 µM (a stock solution of 10 mM in DMSO diluted with growth medium) and with commercial probe for lysosomes labelling (LysoTracker Deep Red, Invitrogen, Waltham, MA, USA) at a concentration of 85 nM for 1 h. Afterwards, cells were stained with a commercial probe for nucleus (Hoechst 33342, Thermo Scientific, Waltham, MA, USA) at a dilution factor of 1000× for 10 min. The cells were then imaged by confocal microscopy (LSM 780 Zeiss, Oberkochen, Germany) with 63× oil immersion objective with a 405 nm laser for Hoechst 33,342 (λ_em_ = 447 nm), a 488 nm laser for the BODIPY derivative (λ_em_ = 530 nm) and a 633 nm laser for LysoTracker Deep Red (λ_em_ = 695 nm).

#### 3.6.2. Quantitative Analysis of Colocalization

Signal thresholds were determined using samples incubated with BODIPY derivative **3** or Lysotracker. Colocalization analysis was carried out using ImageJ with plugin JACoP [46,47] and R statistical Language V4.1.0 (R Core Team, Vienna, Austria). The measurements were repeated at least in three independent images for three different cells. Costes’ automatic threshold was used on the images of each sample to minimize the contribution of sample noise [48]. Pearson’s correlation coefficient, where $R_{i}$ and $G_{i}$ represent red and green channel intensity in each pixel, and $\bar{R}$ and $\bar{G}$ are red and green channel average intensity, was calculated to demonstrate positive spatial correlation. The average and standard deviation of the results were calculated for each condition and result.

(9) $$PCC=\frac{\sum_{i}\left(R_{i}-\bar{R}\right)\times \left(G_{i}-\bar{G}\right)}{\sqrt{\sum_{i}{\left(R_{i}-\bar{R}\right)}^{2}\times \sum_{i}{\left(G_{i}-\bar{G}\right)}^{2}}}$$

## 4. Conclusions

In conclusion, the *meso* anthracene-BODIPY derivative **3** functionalized with a benzimidazole moiety at position 2, as well as the corresponding precursors **1**–**2**, were synthesized and characterized by NMR, UV-Vis, fluorescence and transient absorption spectroscopies and high-resolution mass spectrometry. The effect of pH on the absorption and fluorescence emission of BODIPY derivative **3** was further investigated to discover a significant red shift in the maximum absorption band and a fluorescent enhancement upon pH variation from 8.7 to 1.7, which can be attributed to the protonation of the benzimidazole NH. Moreover, in vitro studies of cancer HeLa cells with BODIPY **3** demonstrated not only its notable cell membrane permeability and biocompatibility, but also its ability to accumulate and label lysosomes. Importantly, these results provide evidence for the potential of the BODIPY derivative as a fluorescent probe to investigate the lysosomal dynamics in living cells.

## Figures and Tables

**Table 1:** Synthesis and photophysical data (including absorption, λ_abs_, and fluorescence emission maxima, λ_fluo_, fluorescence quantum yields, ϕ_F_, Stokes-shift, Δ_SS_, and singlet oxygen sensitization quantum yields, ϕ_Δ_) for the BODIPY derivatives **1**–**3**, in tetrahydrofuran solution and acetonitrile (data in brackets) at 293 K.

Compound	Yield (%)	λ_abs_	λ_fluo_	Δ_SS_	ϕ_F_	ϕ_Δ_
		(nm)	(nm)	(cm^−1^)		
**1**	74	505	515	385	0.43	0.29
		(502)	(515)		(0.03)	
**2**	47	502	525	873	0.02	0.36
		(497)	(509)		(0.01)	
**3**	30	521	585	2100	0.35	0.04
		(515)	(600)		(0.10)	

## Data Availability

Not applicable.

## Supplementary Materials

The following supporting information can be downloaded at: https://www.mdpi.com/article/10.3390/molecules27228065/s1, Synthesis and characterization of BODIPYs 1-3 and Figure S1: Comparison between the absorption and fluorescence emission spectra of anthracene and BODIPY 1 in tetrahydrofuran solutions at room temperature; Figure S2: Normalized absorption and fluorescence emission spectra for the investigated BODIPY derivatives in acetonitrile solution at room temperature.

## Sample Availability

Not available.
