# Supplementary material for: Bioimaging of Lysosomes with a BODIPY pH-Dependent Fluorescent Probe
Source: Molecules. 2022 Nov 20;27(22):8065. doi: 10.3390/molecules27228065 (PMC9696654; doi:10.3390/molecules27228065)
Supplement: Supplementary file 1 [file molecules-27-08065-s001.zip › molecules-2014305-supplementary.pdf]

Supporting Information

Bioimaging of lysosomes with a BODIPY pH-dependent fluorescent probe

Raquel C. R. Gonçalves,[1,2] Efres Belmonte-Reche,[2] João Pina,[3] Milene Costa da Silva,[2] Sónia C. S. Pinto,[1] Juan Gallo,[2] Susana P. G. Costa,[1] M. Manuela M. Raposo*[1]

[1] Centre of Chemistry, University of Minho, Campus de Gualtar, 4710-057 Braga, Portugal.

[2] Advanced (magnetic) Theranostic Nanostructures Lab, International Iberian Nanotechnology Laboratory, Av. Mestre José Veiga s/n 4715-330 Braga, Portugal

[3] CQC-IMS, Department of Chemistry, University of Coimbra, 3004-535 Coimbra, Portugal.

1- Materials and general instrumentation....................S2

2- Synthesis of compounds **1-3**....................S2-S4

3- Figure S1- Comparison between the absorption and fluorescence emission spectra of anthracene and BODIPY **1** in tetrahydrofuran solutions at room temperature S5

4- Figure S2- Normalized absorption and fluorescence emission spectra for the investigated BODIPY derivatives in acetonitrile solution at room temperature S6-S7

5- References S7

1- Materials and general instrumentation

NMR spectra were obtained on a Bruker Avance III 400 at an operating frequency of 400 MHz for ^{1}H and 100.6 MHz for ^{13}C, using the solvent peak as an internal reference (δ relative to TMS). Peak assignments were supported by spin decoupling-double resonance and bidimensional heteronuclear techniques. Mass spectrometry analysis was performed at the "C.A.C.T.I.—*Unidad de Espectrometria de Masas*" at the University of Vigo, Spain. All reagents were purchased from Sigma-Aldrich, Acros and Fluka and used as received. Thin-layer chromatography (TLC) was carried out on 0.25 mm thick precoated silica plates (Merck Fertigplatten Kieselgel $60F_{254}$), and spots were visualized under ultraviolet (UV) light. Chromatography on silica gel was carried out on Merck Kieselgel (230–400 mesh).

The synthesis of BODIPY derivatives **1-3** have been reported recently by our group [1,4], as described below, and by others [2,3].

2- Synthesis of BODIPYs 1-3

Synthesis of BODIPY derivative 1 [1,2]

2,4-Dimethylpyrrole (190 mg, 2.0 mmol) and 9-anthracenecarboxaldehyde (206 mg, 1.0 mmol) were dissolved in dry dichloromethane (100 mL). One drop of trifluoroacetic acid was added and the mixture was allowed to stir for 50 min at room temperature. A solution of 2,3-dichloro-5,6-dicyano-1,4-benzoquinone (454 mg, 2.0 mmol) in dry dichloromethane (100 mL) was added to the mixture. Stirring was continued for another 50 min and then triethylamine (2.27 mL, 16.3 mmol) was added. After stirring for 15 min, $BF_3.OEt_2$ (3.41 mL, 27.6 mmol) was added and further stirred for 30 min. The solvent was evaporated under reduced pressure and the crude residue was purified by dry flash chromatography (petroleum ether/ethyl acetate, 5:1). The pure product was obtained as a red solid (306 mg, 74%.

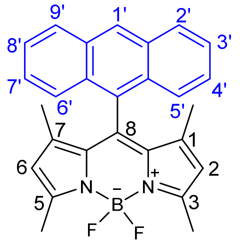

^{1}H NMR (400 MHz, $CDCl_3$): δ =0.67 (s, 6H, CH_3-1 and CH_3-7), 2.65 (s, 6H, CH_3-3 and CH_3-5), 5.91 (s, 2H, H-2 and H-6), 7.43 (dt, *J*=1.2 and 8 Hz, 2H, H-3' and H-8'), 7.50 (dt, *J*=1.2 and 8.4 Hz, 2H, H-4' and H-7'), 7.94 (dd, *J*=0.8 and 8.8 Hz, 2H, H-2' and H-9'), 8.04 (d large, *J*=8.4 Hz, 2H, H-5' and H-6'), 8.59 (s, 1H, H-1') ppm. ^{13}C NMR (100.6 MHz, $CDCl_3$): δ =13.29 (CH_3-C1 and CH_3-C7), 14.67 (CH_3-C3 and CH_3-C5), 121.15 (C2 and C6), 125.07 (C2' and C9'), 125.72 (C4'and C7'), 126.93 (C3'and C8'), 128.20 (C1'a and C9'a), 128.25 (C5' and C6'), 128.32 (C1'), 129.66 (C5'b), 131.28 (C5'a and C6'a), 132.35 (C7a and C8a), 138.94 (C8), 142.87 (C1 and C7), 155.74 (C3 and C5) ppm. MS (ESI) *m/z* (%): 426 ($[M + 2]^{+\bullet}$, 29), 425 ($[M + 1]^{+\bullet}$, 92), 424 ($[M]^{+\bullet}$, 26), 405 (100), 291 (31), 209 (7), 147 (8), 102 (29); HRMS (ESI) *m/z* : $[M + 1]^{+\bullet}$ calcd for $C_{27}H_{24}BF_2N_2$, 425.1995; found 425.1989.

Synthesis of BODIPY derivative 2 [3]

A mixture of *N,N*-dimethylformamide (1.69 mL, 23.1 mmol) and $POCl_3$ (1.70 mL, 18.2 mmol) was stirred for 5 min at 0 °C under N_2. The mixture was allowed to reach room temperature and stirred for additional 30 min. Compound **1** (70 mg, 0.17 mmol) dissolved in dichloroethane (7 mL) was added dropwise while stirring. The reaction mixture was heated for 2 h at 50 °C. After cooling, the solution was poured slowly into 40 mL of saturated sodium bicarbonate solution at 0 °C and stirred for 30 min at room temperature. Ethyl acetate (50 mL) was added to the reaction mixture and the resulting organic layer separated and washed with water (2 × 50 mL). The organic layer was dried with anhydrous $MgSO_4$ and filtered. After evaporation of the solvent to dryness, the crude residue was purified by a silica gel chromatography column, using dichloromethane as eluent. The pure product was obtained as a red solid (36 mg, 47%).

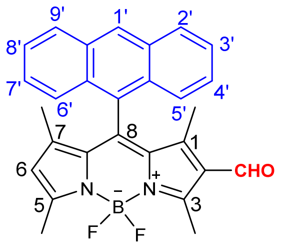

^{1}H NMR (400 MHz, $CDCl_3$): δ = 0.68 (s, 3H, CH_3-7), 0.98 (s, 3H, CH_3-1), 2.71 (s, 3H, CH_3-5), 2.92 (s, 3H, CH_3-3), 6.08 (s, 1H, H-6), 7.46 (dt, *J*=1.6 and 8 Hz, 2H, H-3' and H-8'), 7.53 (dt, *J*=1.6 and 8 Hz, 2H, H-4' and H-7'), 7.84 (dd, *J*=0.8 and 8.4 Hz, 2H, H-2' and H-9'), 8.08 (d large, *J*=8.4 Hz, 2H, H-5' and H-6'), 8.64 (s, 1H, H-1'), 9.91 (s, 1H, CHO) ppm. ^{13}C NMR (100.6 MHz, $CDCl_3$): δ =10.50 (CH_3-

C1), 11.14 (CH_3-C7), 13.23 (CH_3-C3), 13.80 (CH_3-C5), 124.00 (C6), 124.58 (C2' and C9'), 125.95 (C4'and C7'), 126.25 (C2), 127.42 (C3' and C8'), 127.65 (C5'b), 128.58 (C5' and C6'), 128.94 (C1'), 129.52 (C1'a and C9'a), 130.50 (C8a), 131.31 (C5'a and C6'a), 135.13 (C7a), 141.24 (C8), 142.76 (C1), 146.96 (C7), 156.75 (C3), 161.94 (C5), 185.80 (CHO) ppm. MS (ESI) *m/z* (%): 454 ($[M + 2]^{+\bullet}$, 30), 453 ($[M + 1]^{+\bullet}$, 100), 452 ($[M]^{+\bullet}$, 23); HRMS (ESI) *m/z* : $[M + 1]^{+\bullet}$ calcd for $C_{28}H_{24}BF_2N_2O$, 453.1944; found 453.1936.

Synthesis of BODIPY derivative 3 [4]

BODIPY aldehyde **2** (45.2 mg, 0.10 mmol), ethanol (10 mL) and $NaHSO_3$ (10.4 mg, 0.10 mmol) were added to a round bottomed flask and stirred at room temperature for 4 h. Then, dry DMF (5 mL) and *o*-phenylenediamine (8.6 mg, 0.08 mmol) were added and the solution was heated for 2 h at 80 °C. The reaction mixture was cooled to room temperature, ethyl acetate was added (10 mL) and the mixture was washed with water (3 × 10 mL). The organic phase was dried with anhydrous $MgSO_4$ and the solvent was evaporated to dryness. The resulting crude was purified by a silica gel chromatography column using dichloromethane as eluent and pure compound **3** was obtained as a red solid (12 mg, 30%).

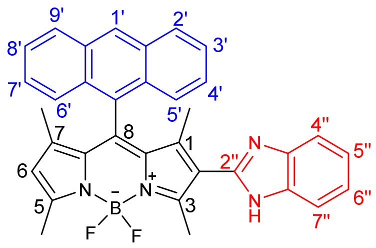

^{1}H NMR (400 MHz, $CDCl_3$): δ =0.69 (s, 3H, CH_3-7), 0.83 (s, 3H, CH_3-1), 2.69 (s, 3H, CH_3-5), 2.84 (s, 3H, CH_3-3), 6.02 (s, 1H, H-6), 7.22-7.27 (m, 2H, H-5'' and H-6''), 7.42 (dt, *J*=1.2 and 8, 2H, H-4' and H-7'), 7.48 (dt, *J*=1.2 and 8, H-3' and H-8'), 7.56 (s large, 2H, H-4'' and H-7'') 7.88 (d, *J*= 8.8, 2H, H-2' and H-9'), 8.03 (d, *J*=8.4, 2H, H-5' and H-6'), 8.59 (s, 1H, H-1') ppm. MS (ESI) *m/z* (%): 542 ($[M + 2]^{+\bullet}$, 39), 541 ($[M + 1]^{+\bullet}$, 100), 540 ($[M]^{+\bullet}$, 24); HRMS (ESI) *m/z* : $[M + 1]^{+\bullet}$ calcd for $C_{34}H_{28}BF_2N_4$, 541.2370; found 541.2369.

Figure S1- Comparison between the absorption and fluorescence emission spectra of anthracene and BODIPY 1 in tetrahydrofuran solutions at room temperature

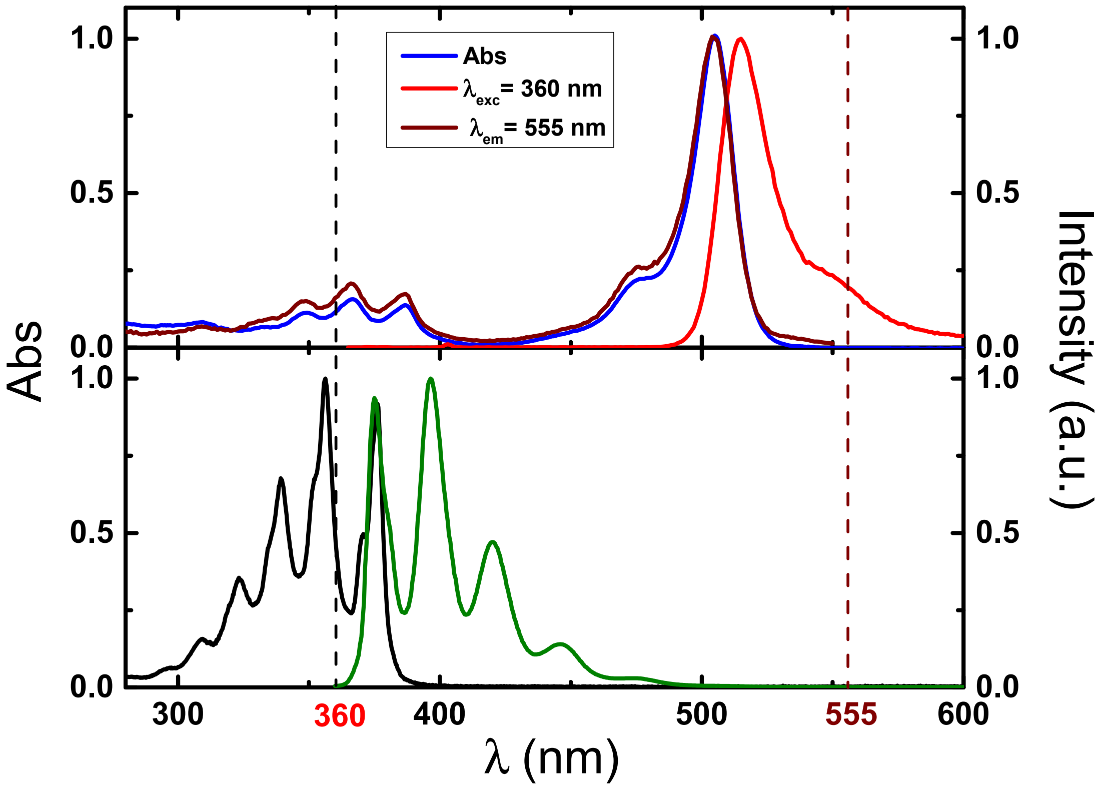

Figure S1- Comparison between the absorption and fluorescence emission spectra of anthracene and BODIPY **1** in tetrahydrofuran solutions at room temperature. Also presented is the fluorescence excitation spectra of BODIPY **1** collected with emission at 555 nm, displaying good agreement with the absorption spectra.

Figure S2- Normalized absorption and fluorescence emission spectra for the investigated BODIPY derivatives in acetonitrile solution at room temperature.

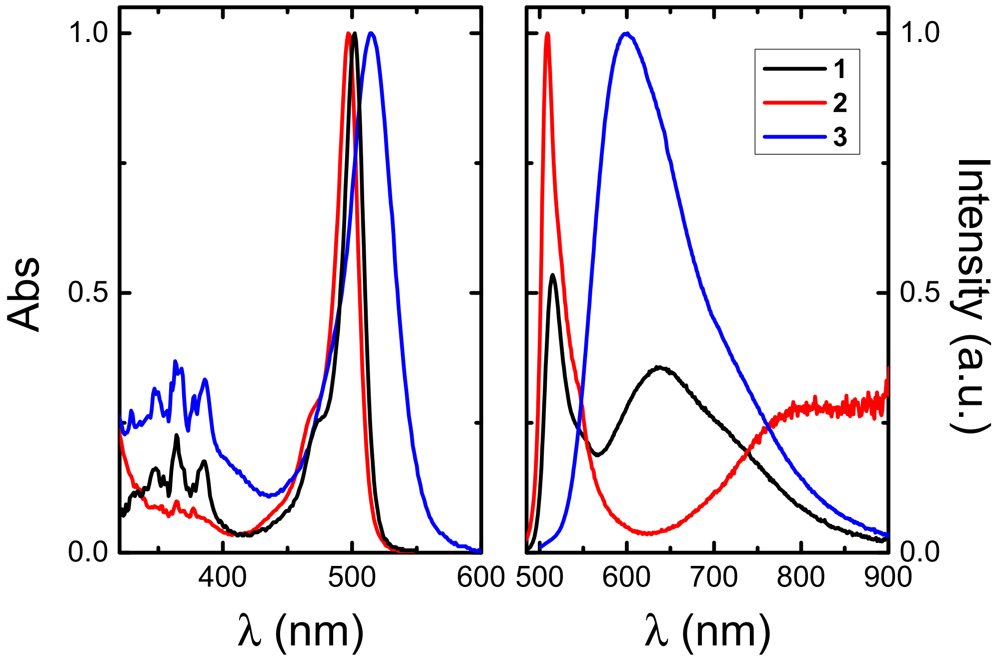

Figure S2- Normalized absorption and fluorescence emission spectra for the investigated BODIPY derivatives in acetonitrile solution at room temperature.

5- References

1. Pinto, S.C.S.; Gonçalves, R.C.R.; Costa, S.P.G.; Raposo, M.M.M. Synthesis and Characterization of a *Meso*-Anthracene-BODIPY Derivative for Colorimetric Recognition of Cu^{2+} and Fe^{3+}. *Chem. Proc.* **2021**, *3*(1), 79, doi: 10.3390/ecsoc-24-08292.
2. Sunahara, H.; Urano, Y.; Kojima, H.; Nagano, T. Design and Synthesis of a Library of BODIPY-Based Environmental Polarity Sensors Utilizing Photoinduced Electron-Transfer-Controlled Fluorescence ON/OFF Switching. *J. Am. Chem. Soc.* **2007**, *129*, 5597–5604, doi:10.1021/ja068551y.
3. Jia, Y.; Pan, Y.; Wang, H.; Chen, R.; Wang, H.; Cheng, X. Highly Selective and Sensitive Polymers with Fluorescent Side Groups for the Detection of Hg 2+ Ion. *Mater. Chem. Phys.* **2017**, *196*, 262–269, doi:10.1016/j.matchemphys.2017.05.001.
4. Gonçalves, R.C.R.; Pinto, S.C.S.; Costa, S.P.G.; Raposo, M.M.M. Anion Colorimetric Chemosensor Based on a Benzimidazole-Functionalized BODIPY Derivative. *Chem. Proc.* **2022**, *8*(1), 90, doi: 10.3390/ecsoc-25-11754.
